# Characterisation of CD154^+^ T cells following *ex vivo* birch allergen stimulation defines a close relationship between T cell subsets in healthy volunteers

**DOI:** 10.1186/1471-2172-14-14

**Published:** 2013-03-22

**Authors:** Karen A Smith, Nicola J Gray, Elizabeth Cheek, Femi Saleh, Jo Lavender, Anthony J Frew, Florian Kern, Michael D Tarzi

**Affiliations:** 1 Brighton and Sussex Medical School, Division of Clinical Medicine, Pathogen-Host-Interactions Group, University of Sussex, Brighton, BN1 9PS, UK; 2 Department of Respiratory Medicine, Royal Sussex County Hospital, Brighton, BN2 5BE, UK; 3 Department of Computing, Engineering and Mathematics, University of Brighton, Brighton, BN2 4GJ, UK; 4 Diagnostic Immunology Laboratory, Blood Sciences Department, Royal Sussex County Hospital, Brighton, BN2 5BE, UK

**Keywords:** Allergen-specific T cell, Birch pollen allergy, *Ex vivo* phenotyping, Flow cytometry

## Abstract

**Background:**

Allergic sensitisation has been ascribed to a dysregulated relationship between allergen-specific Th1, Th2 and regulatory T cells. We hypothesised that the relationship between these T cell subsets could be better defined using a short-term allergen stimulation system followed by direct analysis of CD154-positive T cells. Using peripheral blood samples from birch pollinosis patients and healthy non-atopic controls, we sought to explore the frequencies and phenotype of birch-stimulated CD154-positive T helper cells following *ex vivo* birch allergen stimulation.

**Results:**

Activated CD154-positive Th1, Th2 and Tr1-like cells, that co-expressed IFNγ, IL-4 and IL-10 respectively, were identified in both birch-allergic and non-allergic participants. We observed a close correlation between Th1, Th2 and Tr1-like cell frequency in non-allergic volunteers, such that the three parameters increased together to maintain a low Th2: Th1 ratio. The relationship between Th1, Th2 and Tr1-like responses was dysregulated in birch-allergic patients, with abrogation of the IL-10 response and a higher Th2: Th1 ratio. A close correlation was observed between Th2 cell frequency and the absolute concentration of birch-specific IgE within the birch-allergic group, and we confirmed previous reports of a more differentiated T cell phenotype in allergic subjects.

**Conclusions:**

The findings demonstrate an important balance between IFNγ, IL-4 and IL-10 T cell responses to birch allergen in health, where Th2 responses to allergens were frequently observed, but apparently balanced by Th1 and regulatory responses. The detection of CD154 positive T cells after short-term antigen stimulation may be a useful method for the detection of T cell responses to allergens when cost, speed and convenience are priorities.

## Background

Activated Th2 cells play a key role in the initiation and maintenance of allergic diseases [1]. Over the last decade, the Th2 paradigm has been refined to stress the importance of balance between Th1, Th2 and regulatory responses to allergens in determining the outcome [2]. However, much of the literature relates to cultured cell populations, which may provide only limited information about the true nature of allergen-specific T cells. More recently, class II tetramer methods have become the reference method for the *ex vivo* detection of allergen-specific T cells [3], but this methodology is restricted to immunodominant epitopes in subjects with the appropriate HLA-DR haplotype; furthermore, co-staining for intracellular cytokine expression still implies *in vitro* expansion and/or mitogen stimulation.

The early activation marker CD154 is transiently expressed following ligation of the T cell receptor, providing direct access to an antigen-specific population following *ex vivo* stimulation [4,5]. The method was shown to produce similar results compared to cultured T cell systems following allergen stimulation [6], and has been used by Campbell *et al.* to track T cell responses during experimental ragweed desensitisation [7]. However, detailed phenotyping of CD154^+^ T cells after allergen stimulation has not been reported to date.

We hypothesised that the relationship between Th1, Th2 and regulatory T cells responding to allergen could be better defined using this short-term stimulation system. The objective of this study was to obtain a detailed phenotypic analysis of the allergen-responsive T cells using a panel of surface markers and intracellular cytokines, in patients with birch pollinosis compared to healthy non-atopic control subjects.

## Results

### CD154^+^, Th1, Th2 and Tr1-like populations may be detected after *ex vivo* birch allergen stimulation

**Figure 1:**
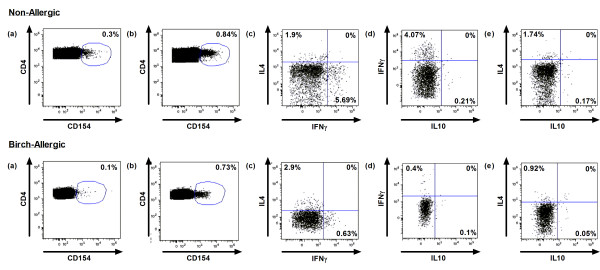
**Example of original data in representative non-allergic and birch-allergic individuals. **Dot plots illustrate CD4^+^CD154 expression in (**a**) unstimulated and (**b**) birch allergen-stimulated PBMC. (**c**) Identification of CD154^+^IFNγ^+^ Th1 population and CD154^+^IL-4^+^ Th2 population. CD154^+^IL-10^+ ^T cells did not co-express (**d**) IFNγ or (**e**) IL-4. Plots illustrate log fluorescence intensity for all markers. Percentages represent background-corrected cytokine expression.

**Figure 2:**
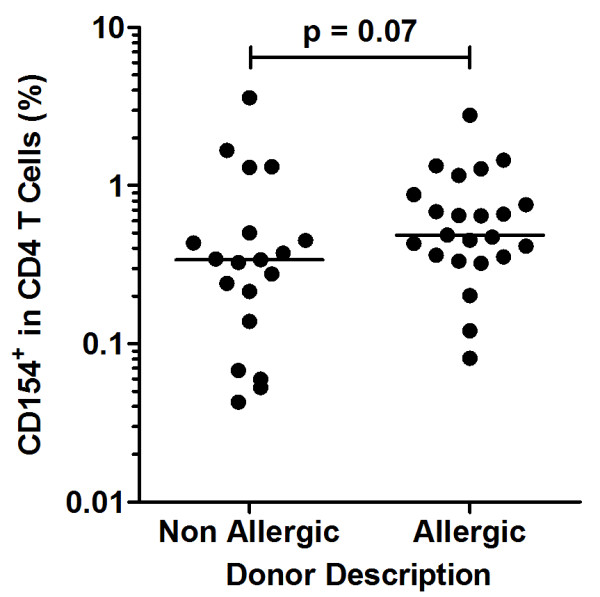
**CD154**^**+ **^**T cells (% of CD4) in non-allergic and birch-allergic participants.**

A CD154^+^ T cell population was detected in both allergic and non-allergic subjects after birch allergen stimulation (Figure 1a, b). Th1 cells were defined as CD154^+^ IFNγ^+^ and Th2 cells as CD154^+^IL-4^+^ (Figure 1c). In addition, we detected a CD154^+^IL-10^+^ population that did not express IFNγ or IL-4 in excess of background signal (Figures 1d, e); for brevity, we refer to this population as Tr1-like. The CD154 single-positive response represented a median of 0.34% and 0.48% of the total CD4 T cell population in non-allergic and birch-allergic participants, respectively (p = 0.07, Figure 2).

### A Th1/ Tr1-like response predominates in birch-tolerant individuals

**Figure 3:**
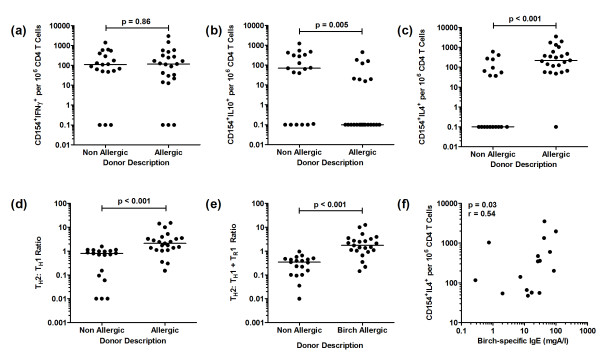
**Birch allergen-induced Th1, Th2 and Tr1-like cell populations in birch-allergic and non-allergic participants. **Data represents (**a**) CD154^+ ^IFNγ^+ ^cells, (**b**) CD154^+^IL-10^+ ^cells and (**c**) CD154^+^IL-4^+ ^cells illustrated as the background-corrected frequency of positive cells per 10^6 ^CD4 T cells on a log scale, (**d**) Th2:Th1 ratio, (**e**) Th2: Th1 + Tr1 ratio and (**f**) correlation of CD154^+^IL-4^+ ^cell frequency and IgE levels in birch-allergic individuals

In the non-allergic group, the predominant T cell response to birch allergen comprised of CD154^+^IFNγ^+^ Th1 cells (Figure 3a). A significantly higher frequency of CD154^+^IL-10^+^ T cells was also identified in non-allergic controls compared to birch-allergic individuals (Figure 3b), although IL-10 was not detected universally. Within the entire birch-allergic group the median frequency of IL-10^+^ T helper cells was significantly lower (p = 0.005), but the distribution was bimodal: fifteen of 23 allergic participants did not produce detectable IL-10, whereas in 8 subjects IL-10^+^ T helper cells were present at a similar frequency compared to the non-allergic group. Further staining of the CD154^+^IL-10^+^ population was performed in three representative non-allergic donors: only 23.5% of the CD154^+^IL-10^+^ cells expressed FoxP3 and only 3.5% were FoxP3^+^CD25^+^CD127^Low^. The IL-10^+^ T cell population was mainly negative for CTLA-4 (7.4%), GITR (18.7%), TGF-β (9.4%) and granzyme A (3.6%), with higher expression of granzyme B (21.1%) and perforin (30.4%).

### The Th2/ Th1 ratio separates birch-allergic and birch-tolerant participants better than the frequency of Th2 and Th1 cells

The CD154^+^IL-4^+^ Th2 response was greater in birch-allergic compared to birch-tolerant participants (p < 0.001, Figure 3c). However, there was considerable overlap between the groups as the Th2 lymphocyte frequency in the non-atopic group was bimodally distributed: IL-4 was not detected in 53% of the group, but in the remaining 47%, the frequency of Th2 lymphocytes was similar to the allergic group. By contrast, CD154^+^IFNγ^+^ Th1 cells were detected in most participants and there was no difference between the groups (Figure 3a).

Despite this overlap, the groups were well-separated on the basis of the Th2: Th1 ratio, which was significantly higher in birch-allergic subjects compared to the non-atopic population (p < 0.001, Figure 3d). The Th2: Th1 + Tr1 ratio was also significantly higher in the birch-allergic group (p < 0.001, Figure 3e).

A close correlation was observed between Th2 cell frequency and the absolute concentration of birch-specific IgE (analysis restricted to sixteen of 23 birch-allergic participants (p = 0.03, r = 0.54) (Figure 3f).

### A correlation between Th1, Th2 and Tr1-like responses in birch-tolerant subjects is dysregulated in birch pollinosis

**Figure 4:**
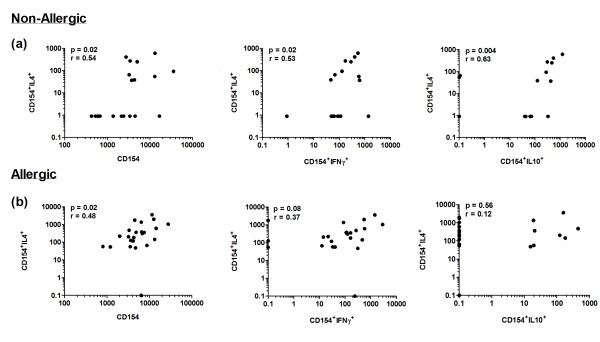
**Correlations between Th2, Th1 and Tr1-like cell populations in (a) non-allergic and (b) birch-allergic individuals. **Data represents the background-corrected frequency of CD154^+^IL-4^+ ^Th2 cells, CD154^+^IFNγ^+^ Th1 cells and CD154^+^IL-10^+ ^Tr1-like cells per 10^6 ^CD4 T cells.

Amongst non-allergic participants, positive correlations were noted between the frequency of Th2 cells and the total CD154 response (p = 0.02, r = 0.54), the Th1 response (p = 0.02, r = 0.53) and the Tr1-like response (p = 0.004, r = 0.63) (Figure 4a). Amongst allergic participants, the relationship between Th2 and Th1 cell frequency was weaker and non-significant (p = 0.08, r = 0.37), and there was no dependence between the frequency of Th2 and Tr1-like lymphocytes (Figure 4b).

### T cell responses to control allergens and seasonal modulation of birch responses support antigen specificity of the assay

We investigated seasonal changes in T cell responses to birch pollen allergen between the peak birch pollen season (April) compared to out of season (August – November) in a small group of allergic and non-allergic participants.

**Figure 5:**
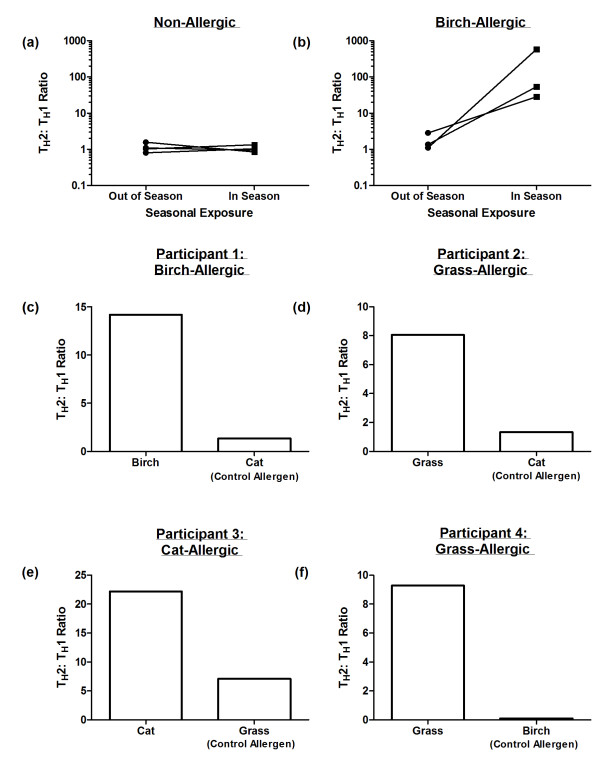
**Supporting evidence in favour of allergen specificity of the CD154**^**+ **^**T cell assay. **Data represents the Th2: Th1 ratio during the peak birch pollen season (April) and out of season (August – November) in (**a**) non-allergic and (**b**) birch-allergic individuals. (**c-f**) Th2: Th1 ratio following stimulation with the test allergen (to which the participant has IgE and clinical symptoms) compared to the Th2: Th1 ratio following stimulation with a control allergen (to which the participant has neither IgE nor clinical symptoms) in four representative participants.

Amongst non-allergic participants, the Th2: Th1 ratio was maintained at low levels both in and out of the birch pollen season (Figure 5a). In contrast, this ratio demonstrates a marked increase during seasonal exposure in three birch-allergic subjects (Figure 5b).

In addition, we characterised CD154^+^ T cell responses under the same experimental conditions following PBMC stimulation with allergens to which atopic patients are not sensitised (Figure 5c). In all cases, the test antigen (to which the participants have IgE and clinical symptoms) elicited a high Th2: Th1 ratio as expected. Following stimulation with a control allergen (to which the participants have neither IgE nor clinical symptoms), the Th2: Th1 ratio was low, and similar to that of the non-allergic control participants.

A subgroup of non-atopic IL-4 ‘responders’ maintain a low Th2: Th1 ratio, whilst a subgroup of allergic IL-10 ‘responders’ produce more birch-specific IgG4

**Figure 6:**
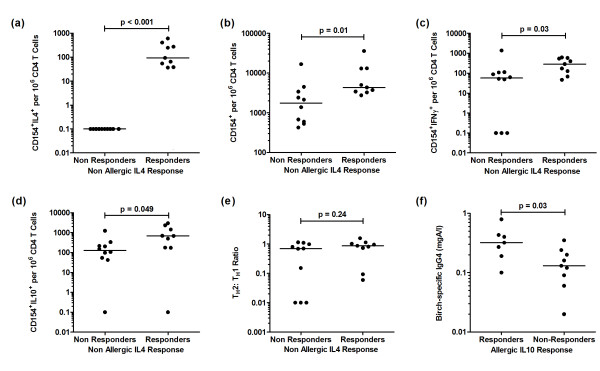
**Background-corrected birch-allergen induced cytokine expression in non-allergics (IL4-responders vs. IL4 non-responders). **Data represents (**a**) CD154^+^IL-4^+^, (**b**) CD154^+^, (**c**) CD154^+^IFNγ^+^, (**d**) CD154^+^IL-10^+ ^illustrated as the frequency of positive cells per 10^6 ^CD4 T cells on a log scale, (**e**) Th2:Th1 ratio and (**f**) IgG4 levels in birch-allergic IL-10 responders vs. IL-10 non-responders.

To further explore the bimodal distribution of Th2 lymphocyte frequencies in non-atopic participants, we defined ‘IL-4 responder’ and ‘IL-4 non-responder’ subgroups for comparison. The frequency of Th1 and Tr1 cells was significantly greater in the IL-4 responder group; both groups maintained a similarly low Th2: Th1 ratio (Figure 6a-e). There was no difference in birch IgG4 concentrations between IL-4 responders and non-responders (data not shown). By contrast, subgroup analysis applied to the allergic population with respect to IL-10 responders/ non-responders demonstrated significantly greater levels of birch IgG4 in the IL-10-producing group (Figure 6f), but did not demonstrate any significant differences in the frequency of Th1 or Th2 cells (data not shown). There were no differences in birch-specific IgE levels between IL10 responder and non-responder allergic subgroups (data not shown).

### Th1 and Th2 cells have a more differentiated phenotype in allergic participants

**Figure 7:**
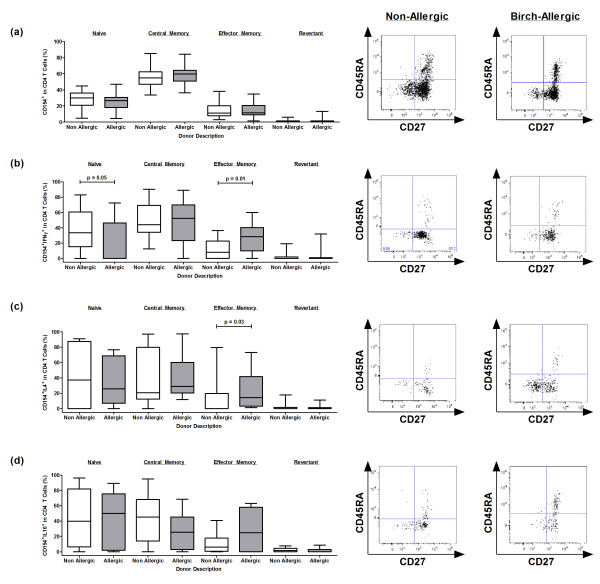
**Phenotypic analysis of birch-stimulated T cell populations based on CD27 and CD45RA cell surface expression. **Data represents the phenotype and representative raw data of (**a**) CD154, (**b**) CD154^+^IFNγ^+^, (**c**) CD154^+^IL-4^+^ and (**d**) CD154^+^ IL-10^+^ T cells responses in non-allergic and birch-allergic individuals, expressed as the background-corrected percentage of positive cells of the total responding CD4 T cell population. CD154+ and CD154 + cytokine + T cell populations were initially calculated using Boolean gating and subsequently analysed for CD27 and/or CD45RA expression.

We also investigated the phenotype of responding CD154^+^ and CD154^+^cytokine^+^ T cell populations following *ex vivo* birch stimulation using the expression of cell surface markers CD27 and CD45RA. Subsets were defined as naive (CD45RA^+^CD27^+^), central memory (CD45RA^-^CD27^+^), effector memory (CD45RA^-^CD27^-^) and effector memory-like short-lived “revertant” (CD45RA^+^CD27^-^) cells (Figure 7). Surprisingly, a proportion of CD154^+^cytokine^+^ T cells expressed markers consistent with a naive phenotype. Further staining in three participants confirmed the co-expression of CCR7 and CD62L in these cells (data not shown). In non-allergic participants, the majority of CD154^+^ and CD154^+^cytokine^+^ T cells were distributed between the naive and central memory compartments. By contrast, effector memory Th1 and Th2 cells were mainly confined to the birch-allergic group.

## Discussion

CD154 has been demonstrated to identify an antigen-specific T cell population following short-term stimulation with pathogen-derived antigens [4,5]. We present here the first detailed description of CD154^+^ T cells responding to birch allergen in non-atopic control subjects and birch pollinosis patients. Activated Th1, Th2 and Tr1-like lymphocytes were identified in varying proportions in allergic and non-allergic participants, with considerable overlap between the groups, supporting the mixed cytokine profile previously described for a number of allergens [8,9]. However, we demonstrate a novel relationship between the frequencies of these T cell subsets in birch tolerance, maintaining a low Th2: Th1 ratio and an appropriate frequency of IL-10^+^ T cells. This relationship was dysregulated in allergy, with the Th2: Th1 ratio maintained at higher levels and the IL-10 response abrogated. Our experiments utilised birch allergen extract antigen rather than Bet v 1, and we have not performed definitive experiments to demonstrate the antigen-specificity or functional properties of the T cell subsets identified. Despite these limitations, numerous aspects of the data support specificity for birch pollen, including: accurate discrimination between allergic and non-allergic participants on the basis of the Th2: Th1 ratio; a low Th2: Th1 ratio amongst allergic volunteers following PBMC stimulation with a control allergen to which they are not sensitised; an increase in the Th2: Th1 ratio during the birch pollen season amongst allergic participants; a correlation between the Th2 cell frequency and the concentration of birch-specific IgE.

The birch allergen extract contains a small amount of LPS, but we have demonstrated that the equivalent concentration in PBS does not produce a CD154 or cytokine response in this assay system (data not shown). A previous study with CD154/ tetramer co-staining after prolonged cell culture and re-stimulation questioned the validity of CD154 as a marker for allergen-specific T cells [10], but these experimental conditions are known to invalidate the assay, which is only reliable after short-term stimulation [6].The close relationship between Th1, Th2 and Tr1-like cells was first reported by Akdis *et al.*[2] using cytokine capture technology. In further experiments, allergen-specific Th2 responses were enhanced by inhibiting the function of Tr1 lymphocytes or increasing Th2 frequency. Our results support the paradigm that it is the relationship between these T cell subsets rather than their absolute numbers that may determine B cell class switching to IgE. Furthermore, we develop the concept by demonstrating a formal correlation between the frequency of Th2 cells and Th1/ Tr1 cells in the non-allergic group. The relationship between these T cell subsets was dysregulated in the birch allergic group, with abrogation of the IL-10 response and a weaker, non-significant correlation between Th2 and Th1 cell frequency at an increased Th2: Th1 ratio. However, the concentration of circulating birch IgE correlated closely with the frequency of IL-4 positive T cells rather than the Th2: Th1 ratio, consistent with the observations of Crack *et al.*[11], suggesting that the absolute Th2 lymphocyte frequency represents an important parameter once sensitisation has occurred.

Interestingly, the Th2 response to birch allergen amongst non-allergic participants displayed a bimodal distribution. We observed a significantly higher frequency of Th1 and Tr1-like lymphocytes in the IL-4 ‘responder’ group, maintaining a low Th2: Th1 + Tr1 ratio, presumably favouring tolerance to birch allergen in the face of a Th2 response. Birch-specific IgG4 levels were similarly low in both groups, although it would be of interest to repeat this measurement in the birch season. Platt-Mills *et al.* described a ‘modified’ Th2 response characterised by IL-4, IL-10 and the production of cat-specific IgG4, in a group of cat-tolerant, heavily exposed individuals [12,13]. Our population was not exposed to birch pollen and we confirmed that sampling occurred throughout the year, without clustering after the pollen seasons. However, it must be noted that the major birch pollen allergen Bet v 1 belongs to the PR-10 panallergen family; the T cell response to native birch allergen is therefore likely to include cells responding to plant-derived Bet v 1 homologs, representing perennial rather than seasonal allergens. The single-epitope detection of tetramer assays clearly differs markedly in this respect.

Regulatory T cells that secrete IL-10 have a confirmed role in tolerance induction during natural allergen exposure [14] and allergen immunotherapy, both conventional [15,16] and experimental [17]. We confirm in our experiments a higher frequency of IL-10-producing T cells in non-allergic individuals. The IL-10^+^ T cells identified in this assay were predominantly Foxp3-negative. This is consistent with previous reports of inducible FOXP3-negative, CD4+ T cells of regulatory function [18,19]. Although we have not demonstrated the functional suppressive activity of these cells, our findings are similar to previous reports and support a role for these cells in natural tolerance [1,2,20].

IL-10 is known to modulate the function of cells involved in allergic inflammation [21], but the exact mechanisms by which IL-10-secreting T cells exert their regulatory function is not entirely clear. The allergen-specific IL-10-secreting T cells of Akdis *et al.*[2] appeared to operate by a mechanism that was dependent upon PD-1 and CTLA-4 in addition to IL-10. Circulating IL-10-secreting T cells responding to islet cell antigens have been identified in healthy non-diabetic blood donors [22], and operate by perforin and granzyme-mediated killing of antigen presenting cells [23]. It would be interesting to determine whether environmental allergens are subject to similar regulatory mechanisms given the moderate expression of these molecules by Tr1-like cells in our experiments. It is also notable that IL-10 production was bimodally distributed in the allergic population. The frequencies of Th1 and Th2 cells after *ex vivo* birch stimulation were similar between these groups, but the levels of birch-specific IgG4 were greater amongst IL-10 responders. The production of allergen-specific IgG4 is known to be IL-4 and IL-10-dependent [24], and has attracted intense interest over recent years for its role in immunotherapy mechanisms [25]. The clinical significance of our observation is unclear, as the two allergic subpopulations did not appear to differ in disease phenotype.

In tetramer studies, allergen-specific T cells were recently described as late-differentiated in subjects allergic to alder [1] and birch pollen [26], whereas responses to perennial allergens were early-differentiated in both allergic and non-allergic subjects [8,9]. Using conventional classification criteria based on surface expression of CD45RA and CD27, we can confirm that effector memory Th1 and Th2 cell responses to birch allergen are predominantly a feature of birch pollinosis, but responding T cells with an early-differentiated phenotype were observed at high frequency in both allergic and non-allergic participants. As discussed, Bet v 1 may have some characteristics of a perennial allergen in this assay system, possibly explaining the partial agreement with tetramer studies. It was interesting to observe that effector cytokines were readily detected in apparently naive T cells, supported by co-staining for CD62L and CCR7. This is unlikely to reflect non-specific staining of the assay as the frequency is background-corrected following Boolean gating of cytokine-positive populations and CD27/CD45RA analysis. Previous reports have described the ability of naive T cells to acquire memory characteristics, including up-regulation of activation markers and/or effector cytokine activity [27]. On this basis, the apparently naive population may actually represent a relatively undifferentiated memory population, capable of secreting cytokines, but maintaining a naive-like phenotype [28].

## Conclusions

In conclusion, this is the first description of CD154^+^ T cell responses to birch allergen in an *ex vivo* stimulation system. We demonstrate a close relationship between Th1, Th2 and Tr1-like responses to birch allergen in health that conspires to maintain a low Th2: Th1 ratio. In birch-allergic individuals, the relationship is dysregulated with a higher Th2: Th1 ratio, defective Tr1-like responses and IgE concentration that correlates with the frequency of Th2 cells. Direct phenotyping of CD154^+^ T cells after short-term allergen stimulation may be a useful method for the investigation of allergen-specific T cells, particularly where speed, convenience and cost are important considerations.

## Methods

### Study participants

This study was approved by the National Research Ethics Service (South East Coast, Brighton and Hove) and the University of Sussex Ethics Research Committee. All volunteers provided informed written consent. Birch-allergic patients (n = 23) with a history of spring rhinitis +/- pollen food syndrome (n = 18) and a positive skin prick test to birch pollen allergen extract were recruited from the Allergy Clinic of the Royal Sussex County Hospital (Table 1). An age-matched comparison population (n = 19) with no history of atopic diseases and negative skin prick tests to common aeroallergens (birch pollen, grass pollen mix, early pollinating tree pollen mix, mid-pollinating tree mix, cat dander) was recruited from staff and students at Brighton and Sussex Medical School. The study was approved by the National Research Ethics Service (South East Coast, Brighton and Hove) and by the University of Sussex Research Ethics Committee. Blood samples were taken a minimum of eight weeks outside the birch pollen season. For the investigation of seasonal variation in T cell responses to birch allergen, blood samples were taken during the peak birch pollen season (April) and again between August and November.

**Table 1:** Participant demographics

	**Non-allergic**	**Birch-allergic**
**No. of participants**	19	23
**Age**	30 ± 7	37 ± 15
**Sex (M:F)**	10:9	5:18
**Allergic diseases (%):**		
**Asthma**	0	52.2
**Atopic dermatitis**	0	21.7
**Rhinitis:**		
**Seasonal**	0	100
**Perennial**	0	34.8
**Cat-Induced**	0	65.2
**Pollen food syndrome**	0	78
**SPT-positive (%):**		
**Birch**	0	100
**Grass**	0	82.6
**Cat**	0	73.9
**House dust mite**	0	47.8
**Moulds**	0	4.4
**Birch-specific IgE (k/Ul)**	0	31.4 ± 29.9

### Antigens

Birch pollen, grass pollen and cat dander allergen extracts was a kind gift of Dr Helga Kahlert (AllergoPharma, Reinbek, Germany). The freeze-dried lyophillisate was reconstituted with phosphate buffered saline (PBS) to 2×10^5^ protein nitrogen units/ml (PNU/ml) (1330 μg/ml) and filtered before storage at -20°C. The final LPS concentration in the stimulation system was estimated at 1.54 ng/ml by LAL test (Pyrochrome C180, Pyroquant Diagnostik, Germany). Phytohaemagglutinin (PHA) (Sigma Aldrich, Dorset, UK) was reconstituted in PBS to 1 mg/ml before storage at -20°C.

### Allergen stimulation

**Figure 8:**
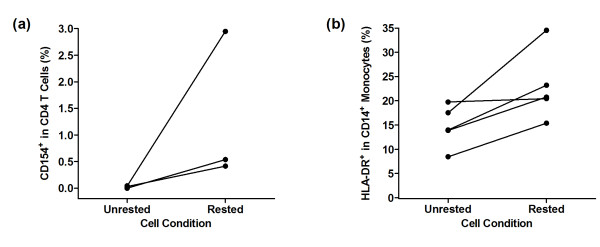
**Assay Optimisation. **(**a**) CD154 expression after birch allergen stimulation in rested vs. unrested PBMC in three representative non-allergic participants. (**b**) HLA-DR expression illustrated as a percentage of total CD14^+ ^monocytes in rested vs. unrested cell populations in five representative non-allergic participants.

Human peripheral blood mononuclear cells (PBMC) were isolated from citrated blood by density centrifugation using Ficoll Paque Plus (GE Healthcare, Buckinghamshire, UK), washed twice in PBS then resuspended at 5 × 10^6^/ml in RPMI 1640 (10% FCS, 2 mM L-Glutamine, 1% Penicillin-Streptomycin). PBMC were then rested for 24 hours at 37°C and 5% humidified CO_2_ to improve allergen-induced CD154 expression, possibly relating to upregulation of CD14^+^ monocyte MHCII expression (Figure 8). After resting, 2 × 10^6^ PBMC were stimulated with allergen at 1000PNU/ ml in a total volume of 500 μl, in 5 ml polypropylene tubes at 37°C and 5% humidified CO_2_ for 2 hours, before the addition of 20 μg/ml Brefeldin A in 500 μl (10 μg/ml final concentration, Sigma Aldrich, Dorset, UK) for the final 14 hours. Unstimulated PBMC served as a negative control for background correction. 10 μg/ml PHA (Sigma Aldrich, Dorset, UK) was used as a positive control.

### Antibody staining

Cells were washed in FACS buffer (PBS, 0.5% BSA, 0.1% sodium azide). Surface staining was performed by incubation for 30 minutes with various combinations of anti-human CD3-Alexafluor700, CD4-PerCP, CD27-FITC, CCR7-AlexaFluor647 (BD Pharmingen, Oxford, UK), CD25-APC-Cy7, CD62L-PacificBlue, CD127-PacificBlue, TGFβ-PeCy7, GITR-PeCy5 (Biolegend, Cambridge, UK), CD45RA-ECD (IQ Products, DL Groningen, The Netherlands), CD4-ECD (Beckman Coulter, High Wycombe, UK), CTLA-4-PE (eBioscience, Hatfield, UK) and Aqvid dead cell stain (Invitrogen, Paisley, UK). Cells were then washed in FACS buffer, fixed and permeabilised (lyse and permeabilisation II solutions, BD Pharmingen). Intracellular staining was performed for 30 minutes with various combinations of anti-human IL-10-PE, IFNγ-PeCy7 (BD Pharmingen), CD154-APC-Cy7, CD154-PacificBlue, IL-4-PE, Granzyme A-PerCP, Granzyme B-Alexafluor647 and Perforin-PacificBlue and IL-10-APC (Biolegend). For analysis of FoxP3 expression, cells were additionally permeabilised using the human FoxP3 buffer set (BD Pharmingen) before intracellular staining with FoxP3-AlexaFluor488 (BD Pharmingen). Fluorochrome-conjugated beads were used for compensation. Fluorescence-minus-one (FMO) controls were performed to ensure the compatibility of fluorochromes and to eliminate antibody effects in cytokine expression.

### Flow cytometric analysis

Cells were washed with FACS buffer prior to acquisition by LSR II flow cytometer (BD Biosciences, Oxford, UK) with FACSDiva. A minimum of 4 × 10^5^ CD4 events were gated for each subject. Aqvid staining demonstrated cell viability close to 100%, therefore live-dead exclusion was not routinely performed. Data were analysed using FlowJO software v9.9.3 (Treestar, Ashland, US). Boolean gating combinations were computed for cytokine and cell marker analysis.

### Birch-specific IgE and IgG4

Serum samples were frozen at -80°C before batch analysis for birch IgE and IgG4 concentration, using a Phadia 100 instrument (Thermofisher IDD, Uppsala, Sweden).

### Statistical analysis

Statistical analysis was supported by Graphpad Prism v5.03 (La Jolla, USA). The data distribution was non-parametric according to the D’Agostino and Pearson omnibus normality test. Median values were used for comparison throughout. All cell frequency values were background-corrected by subtraction of the unstimulated cell frequency from the stimulated cell frequency. Statistical significance was calculated using the two-tailed Mann–Whitney U test with a significance level of 0.05. Spearman rank correlation analysis was used to investigate statistical dependence between variables. To calculate IL-4: IFNγ ratio, the frequency of CD154^+^IL-4^+^ cells was divided by the frequency of CD154^+^IFNγ^+^ cells. Where participants had no detectable CD154^+^IFNγ^+^ or CD154^+^IL-4^+^ cells, a value was defined by allocating a predicted frequency value based on the regression equation from all responding participants.

## Abbreviations

FMO: Fluorescence minus one; LPS: Lipopolysaccharide; MHC: Major histocompatibility complex; PBMC: Peripheral blood mononuclear cells; PBS: Phosphate buffered Saline; PHA: Phytohaemagglutinin; PNU: Protein nitrogen units; RPMI: Roswell park memorial institute.

## Competing interests

The authors declare that they have no competing interests.

## Authors’ contributions

KS carried out the PBMC preparations, birch allergen stimulation and antibody experiments, flow cytometric analysis and helped to draft the manuscript. NG carried out the recruitment of participants and PBMC preparations. EC conducted the statistical analysis. FS and JL performed the serum birch-specific IgG4 and IgE experiments and analysis. AF and FK provided technical assistance and contributed to study design, coordination and participant recruitment. MT conceived the study, coordinated the study design, experimental and statistical analysis and participant recruitment. All authors read and approved the final manuscript.
